# 4-{2-[4-(Dimethyl­amino)­phen­yl]ethen­yl}-1-methyl­pyridinium 4-nitro­benzene­sulfonate

**DOI:** 10.1107/S1600536812009300

**Published:** 2012-03-10

**Authors:** Liang Li, Yiqiang Dai, Yun Jin, Huai Yang, Zhou Yang

**Affiliations:** a Department of Materials Physics and Chemistry, School of Materials Science and Engineering, University of Science and Technology Beijing, Beijing 100083, People’s Republic of China

## Abstract

The asymmetric unit of the title salt, C_16_H_19_N_2_^+^. C_6_H_4_NO_5_S^−^, consists of two cations and two anions. The crystal structure is stabilized by π–π inter­actions between the pyridyl and phenyl rings of the cations, with a centroid–centroid distance of 3.7323 (6) Å.

## Related literature

The title compound was synthesized as part of our continuing research on the non-linear optical properties of DAS (4-*N*,*N*-dimethyl­amino-4′-*N*′-methyl­stilbazolium) derivatives. For the synthesis, see: Okada *et al.* (1990 ▶). For background to non-linear optical materials, see: Yang *et al.* (2005 ▶); Kumar *et al.* (2009 ▶); Kwon *et al.* (2010 ▶). For the effects of different substit­uents of benzene sulfonate on its non-linear optical properties, see: Ogawa *et al.* (2008 ▶); Okada *et al.* (2003 ▶); Yang *et al.* (2007 ▶); Yin *et al.* (2012 ▶); Li *et al.* (2012 ▶). For standard bond-lengths, see: Allen *et al.* (1987 ▶).
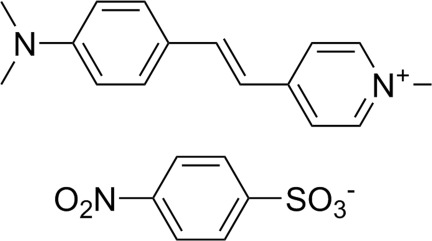


## Experimental

### 

#### Crystal data

C_16_H_19_N_2_^+^·C_6_H_4_NO_5_S^−^
*M* *_r_* = 441.49
Monoclinic, 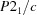

*a* = 18.901 (3) Å
*b* = 6.4504 (10) Å
*c* = 34.222 (6) Å
β = 96.77 (3)°
*V* = 4143.1 (12) Å^3^
*Z* = 8
Mo *K*α radiation
μ = 0.20 mm^−1^
*T* = 173 K
0.20 × 0.16 × 0.13 mm

#### Data collection

Rigaku Saturn 724+ diffractometer
Absorption correction: multi-scan (*CrystalClear*; Rigaku, 2008 ▶) *T*_min_ = 0.752, *T*_max_ = 1.000
20075 measured reflections
9459 independent reflections
7630 reflections with *I* > 2σ(*I*)
*R*_int_ = 0.048

#### Refinement

*R*[*F* ^2^ > 2σ(*F* ^2^)] = 0.075
*wR*(*F* ^2^) = 0.176
*S* = 1.15
9459 reflections
593 parameters
69 restraints
H-atom parameters constrained
Δρ_max_ = 0.42 e Å^−3^
Δρ_min_ = −0.32 e Å^−3^

### 

Data collection: *CrystalClear* (Rigaku, 2008 ▶); cell refinement: *CrystalClear*; data reduction: *CrystalClear*; program(s) used to solve structure: *SHELXS97* (Sheldrick, 2008 ▶); program(s) used to refine structure: *SHELXL97* (Sheldrick, 2008 ▶); molecular graphics: *Mercury* (Macrae *et al.*, 2006 ▶); software used to prepare material for publication: *SHELXL97*.

## Supplementary Material

Crystal structure: contains datablock(s) I, New_Global_Publ_Block. DOI: 10.1107/S1600536812009300/ez2278sup1.cif

Supplementary material file. DOI: 10.1107/S1600536812009300/ez2278Isup2.cdx

Structure factors: contains datablock(s) I. DOI: 10.1107/S1600536812009300/ez2278Isup3.hkl

Additional supplementary materials:  crystallographic information; 3D view; checkCIF report

## Figures and Tables

**Fig. 1.**
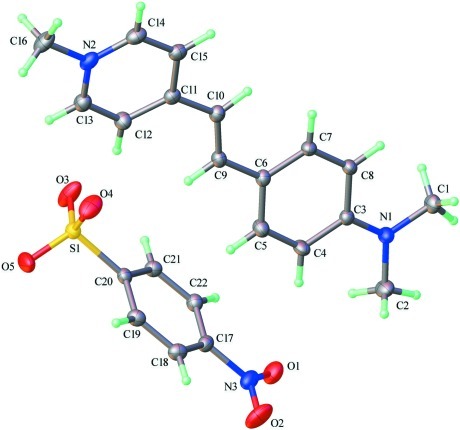
The molecular structure of the title compound showing 50% probability displacement ellipsoids.

**Fig. 2.**
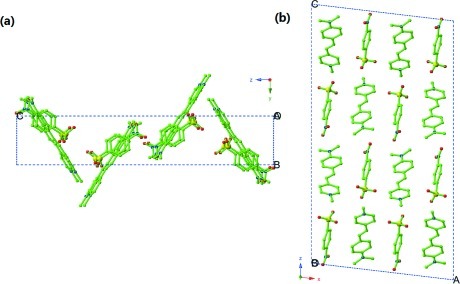
Projections of the crystal packing of the title compound along the (a) x and (b) y axes.

## supplementary crystallographic information

### Comment

Nonlinear optical materials have recently invoked a large amount of interest due to their potential application in harmonic generation, optical information processing, optical storage and two photon pumped lasers (Yang *et al.*, 2005; Kumar *et al.*, 2009; Kwon *et al.*, 2010). The synthesis and crystal growth of the title compound is part of our series of studies on the nonlinear optical properties of DAS (4-N, *N*-dimethylamino-4'-*N*'-methyl-stilbazolium) derivatives (Yang, Mutter *et al.*, 2007; Yin *et al.*, 2012; Li *et al.*, 2012). By changing the anion from 3-nitrobenzenesulfonate to 4-nitrobenzenesulfonate, the space group has changed from monoclinic *P*2_1_ (Ogawa *et al.*, 2008; Okada *et al.*, 2003) to the centrosymmetric space group monoclinic *P*2_1_/*c*. Fig. 1 illustrates the molecular structure of the title salt together with the atomic numbering scheme. The asymmetric unit of the title salt consists of two 4-{2-[4-dimethylamino)phenyl]ethenyl}-1- methylpyridinium cations and two 4-nitrobenzenesulfonate anions. The bond distances and angles in both the cation and anion are in normal ranges (Allen *et al.*, 1987).

The crystal structure is stabilized by a π-π interaction between the pyridyl and C3—C8 phenyl rings with a centroid-centroid distance of 3.7323 (6) Å. The packing diagram of the title salt obtained from X-ray analysis is presented in Fig. 2. Disorder was observed in one of the anions, whereas the structures of the cations were determined unequivocally. The crystallographic data suggests that coulombic interactions between cations and anions play a key role in crystal packing and orientation of the chromophores.

### Experimental

4-{2-[4-(Dimethylamino)phenyl]ethenyl}-1-methylpyridinium 4-nitrobenzenesulfonate was prepared by the metathesization of 4-*N*,*N*-dimethylamino-4'-*N*'-methyl-stilbazolium iodide (Okada *et al.*, 1990) with the sodium salt of the 4-nitrobenzenesulfonic acid. The title salt was then recrystallized from methanol to get high purity material for crystal growth. 4-{2-[4-(Dimethylamino)phenyl]ethenyl}-1-methylpyridinium 4-nitrobenzenesulfonate: yield 79%; ^1^H-NMR (300 MHz, DMSO-d_6_): 8.69 (d, 2H, J= 6.6 Hz, C_5_H_4_N), 8.21 (d, 2H, J= 9.0 Hz, C_6_H_4_SO_3_^-^), 8.04 (d, 2H, J= 6.3 Hz, C_5_H_4_N), 7.93 (d, 1H, J= 16.2 Hz, CH), 7.84 (d, 2H, J= 9.0 Hz, C_6_H_4_SO_3_^-^),7.60 (d, 2H, J= 8.7 Hz, C_6_H_4_SO_3_^-^), 7.19 (d, 1H, J= 16.2 Hz, CH), 6.80(d, 2H, J= 8.7 Hz, C_6_H_4_), 4.17 (s, 3H, NMe), 3.02 (s, 6H, NMe_2_). C, H, N analysis calcd. for C_22_H_23_N_3_O_5_S: C 59.85, H 5.25, N 9.52; found: C 59.89, H 5.32, N 9.59. Crystals were obtained by the slow cooling method from 45°C to room temperature in methanol.

### Refinement

All H atoms were located geometrically (methyl C—H = 0.98 Å and aromatic C—H = 0.95 Å) and refined using a riding model, with *U*_iso_(H) = 1.2 or 1.5*U*_eq_(C).

### Crystal data

**Table:**

C_16_H_19_N_2_^+^·C_6_H_4_NO_5_S^−^	*F*(000) = 1856
*M**_r_* = 441.49	*D*_x_ = 1.416 Mg m^−^^3^
Monoclinic, *P*2_1_/*c*	Mo *K*α radiation, λ = 0.71073 Å
*a* = 18.901 (3) Å	Cell parameters from 11440 reflections
*b* = 6.4504 (10) Å	θ = 1.2–27.5°
*c* = 34.222 (6) Å	µ = 0.20 mm^−^^1^
β = 96.77 (3)°	*T* = 173 K
*V* = 4143.1 (12) Å^3^	Block, red
*Z* = 8	0.20 × 0.16 × 0.13 mm

### Data collection

**Table:**

Rigaku Saturn 724+ diffractometer	9459 independent reflections
Radiation source: Rotating Anode	7630 reflections with *I* > 2σ(*I*)
Confocal monochromator	*R*_int_ = 0.048
Detector resolution: 28.5714 pixels mm^-1^	θ_max_ = 27.5°, θ_min_ = 1.1°
ω scans at fixed χ = 45°	*h* = −23→24
Absorption correction: multi-scan (*CrystalClear*; Rigaku, 2008)	*k* = −8→4
*T*_min_ = 0.752, *T*_max_ = 1.000	*l* = −42→44
20075 measured reflections	

### Refinement

**Table:**

Refinement on *F*^2^	Primary atom site location: structure-invariant direct methods
Least-squares matrix: full	Secondary atom site location: difference Fourier map
*R*[*F*^2^ > 2σ(*F*^2^)] = 0.075	Hydrogen site location: inferred from neighbouring sites
*wR*(*F*^2^) = 0.176	H-atom parameters constrained
*S* = 1.15	*w* = 1/[σ^2^(*F*_o_^2^) + (0.0574*P*)^2^ + 2.7972*P*] where *P* = (*F*_o_^2^ + 2*F*_c_^2^)/3
9459 reflections	(Δ/σ)_max_ < 0.001
593 parameters	Δρ_max_ = 0.42 e Å^−^^3^
69 restraints	Δρ_min_ = −0.32 e Å^−^^3^

### Special details

**Table:**

Geometry. All e.s.d.'s (except the e.s.d. in the dihedral angle between two l.s. planes) are estimated using the full covariance matrix. The cell e.s.d.'s are taken into account individually in the estimation of e.s.d.'s in distances, angles and torsion angles; correlations between e.s.d.'s in cell parameters are only used when they are defined by crystal symmetry. An approximate (isotropic) treatment of cell e.s.d.'s is used for estimating e.s.d.'s involving l.s. planes.
Refinement. Refinement of *F*^2^ against ALL reflections. The weighted *R*-factor *wR* and goodness of fit *S* are based on *F*^2^, conventional *R*-factors *R* are based on *F*, with *F* set to zero for negative *F*^2^. The threshold expression of *F*^2^ > σ(*F*^2^) is used only for calculating *R*-factors(gt) *etc*. and is not relevant to the choice of reflections for refinement. *R*-factors based on *F*^2^ are statistically about twice as large as those based on *F*, and *R*- factors based on ALL data will be even larger.

### Fractional atomic coordinates and isotropic or equivalent isotropic displacement parameters (Å^2^)

**Table:**

	*x*	*y*	*z*	*U*_iso_*/*U*_eq_	Occ. (<1)
S1	0.37863 (3)	0.12715 (9)	0.308633 (19)	0.02941 (17)	
S2	0.12093 (4)	0.62094 (10)	0.17599 (2)	0.03390 (18)	
O8	0.1528 (6)	0.7557 (9)	0.20680 (16)	0.064 (3)	0.370 (8)
O9	0.0563 (4)	0.526 (2)	0.1802 (3)	0.093 (4)	0.370 (8)
O10	0.1751 (5)	0.4736 (14)	0.1683 (2)	0.062 (3)	0.370 (8)
O8'	0.0890 (4)	0.7475 (6)	0.20394 (11)	0.0686 (19)	0.630 (8)
O9'	0.0757 (3)	0.4454 (6)	0.16520 (14)	0.0523 (13)	0.630 (8)
O10'	0.1927 (2)	0.5691 (12)	0.1848 (2)	0.087 (2)	0.630 (8)
O1	0.37877 (13)	0.8246 (4)	0.45206 (7)	0.0559 (6)	
O2	0.41484 (16)	0.5583 (4)	0.48663 (7)	0.0702 (8)	
O3	0.35623 (16)	0.2650 (3)	0.27682 (6)	0.0636 (7)	
O4	0.32662 (14)	−0.0268 (4)	0.31472 (7)	0.0609 (7)	
O5	0.44820 (12)	0.0429 (4)	0.30699 (8)	0.0669 (8)	
O6	0.08242 (19)	1.0636 (4)	−0.00120 (7)	0.0840 (10)	
O7	0.11956 (14)	1.3255 (3)	0.03349 (7)	0.0550 (6)	
N1	0.12972 (12)	0.7625 (3)	0.43882 (7)	0.0346 (5)	
N2	0.13677 (14)	−0.6462 (3)	0.27136 (6)	0.0357 (5)	
N3	0.39469 (14)	0.6409 (4)	0.45540 (7)	0.0416 (6)	
N4	0.36366 (12)	1.2762 (3)	0.04493 (7)	0.0345 (5)	
N5	0.37284 (14)	−0.1338 (3)	0.21319 (6)	0.0378 (6)	
N6	0.10301 (14)	1.1431 (4)	0.03000 (7)	0.0415 (6)	
C1	0.06152 (15)	0.8609 (4)	0.43954 (9)	0.0396 (7)	
H1A	0.0377	0.8733	0.4126	0.059*	
H1C	0.0683	0.9992	0.4512	0.059*	
H1B	0.0321	0.7771	0.4552	0.059*	
C2	0.19048 (16)	0.8511 (5)	0.46267 (9)	0.0438 (7)	
H2B	0.2083	0.7527	0.4833	0.066*	
H2C	0.1762	0.9800	0.4747	0.066*	
H2A	0.2282	0.8808	0.4461	0.066*	
C3	0.13599 (13)	0.5801 (4)	0.41936 (7)	0.0276 (5)	
C4	0.20215 (14)	0.4821 (4)	0.41864 (8)	0.0357 (6)	
H4	0.2434	0.5400	0.4332	0.043*	
C5	0.20844 (15)	0.3036 (4)	0.39728 (8)	0.0371 (6)	
H5	0.2542	0.2434	0.3970	0.045*	
C6	0.14965 (14)	0.2081 (4)	0.37603 (7)	0.0287 (5)	
C7	0.08349 (14)	0.3022 (4)	0.37776 (7)	0.0294 (5)	
H7	0.0421	0.2402	0.3641	0.035*	
C8	0.07638 (13)	0.4817 (4)	0.39858 (8)	0.0307 (6)	
H8	0.0304	0.5407	0.3990	0.037*	
C9	0.15916 (14)	0.0206 (4)	0.35363 (8)	0.0306 (6)	
H9	0.2068	−0.0242	0.3525	0.037*	
C10	0.10748 (14)	−0.0953 (4)	0.33444 (8)	0.0315 (6)	
H10	0.0596	−0.0520	0.3352	0.038*	
C11	0.11937 (14)	−0.2824 (4)	0.31252 (7)	0.0304 (5)	
C12	0.18615 (15)	−0.3475 (4)	0.30397 (8)	0.0354 (6)	
H12	0.2271	−0.2661	0.3123	0.042*	
C13	0.19324 (16)	−0.5275 (4)	0.28375 (8)	0.0371 (6)	
H13	0.2393	−0.5696	0.2784	0.045*	
C14	0.07128 (16)	−0.5876 (4)	0.27859 (8)	0.0389 (7)	
H14	0.0314	−0.6719	0.2696	0.047*	
C15	0.06104 (15)	−0.4083 (4)	0.29873 (8)	0.0355 (6)	
H15	0.0143	−0.3690	0.3034	0.043*	
C16	0.1471 (2)	−0.8361 (4)	0.24863 (9)	0.0482 (8)	
H16B	0.1080	−0.9330	0.2512	0.072*	
H16C	0.1924	−0.9011	0.2587	0.072*	
H16A	0.1476	−0.8004	0.2208	0.072*	
C17	0.39135 (14)	0.5143 (4)	0.41939 (8)	0.0326 (6)	
C18	0.41112 (15)	0.3080 (4)	0.42251 (8)	0.0356 (6)	
H18	0.4262	0.2476	0.4474	0.043*	
C19	0.40826 (14)	0.1920 (4)	0.38835 (8)	0.0338 (6)	
H19	0.4221	0.0504	0.3896	0.041*	
C20	0.38529 (13)	0.2818 (4)	0.35221 (7)	0.0285 (5)	
C21	0.36631 (14)	0.4897 (4)	0.34995 (8)	0.0313 (6)	
H21	0.3515	0.5510	0.3251	0.038*	
C22	0.36883 (14)	0.6077 (4)	0.38385 (8)	0.0331 (6)	
H22	0.3554	0.7497	0.3827	0.040*	
C23	0.30478 (16)	1.3508 (5)	0.01807 (10)	0.0466 (8)	
H23A	0.2952	1.2532	−0.0039	0.070*	
H23C	0.3167	1.4869	0.0079	0.070*	
H23B	0.2624	1.3630	0.0319	0.070*	
C24	0.43187 (15)	1.3785 (4)	0.04490 (9)	0.0389 (6)	
H24B	0.4532	1.4007	0.0721	0.058*	
H24C	0.4250	1.5123	0.0315	0.058*	
H24A	0.4635	1.2915	0.0311	0.058*	
C25	0.35836 (13)	1.0924 (4)	0.06437 (7)	0.0287 (5)	
C26	0.41646 (14)	1.0084 (4)	0.08882 (8)	0.0317 (6)	
H26	0.4609	1.0787	0.0912	0.038*	
C27	0.41043 (14)	0.8274 (4)	0.10934 (7)	0.0298 (5)	
H27	0.4510	0.7751	0.1253	0.036*	
C28	0.34625 (14)	0.7180 (4)	0.10732 (7)	0.0296 (5)	
C29	0.28885 (14)	0.7994 (4)	0.08273 (8)	0.0351 (6)	
H29	0.2446	0.7281	0.0803	0.042*	
C30	0.29433 (14)	0.9806 (4)	0.06174 (8)	0.0345 (6)	
H30	0.2540	1.0304	0.0452	0.041*	
C31	0.33848 (14)	0.5268 (4)	0.12887 (8)	0.0314 (6)	
H31	0.2922	0.4675	0.1269	0.038*	
C32	0.39066 (15)	0.4265 (4)	0.15121 (8)	0.0327 (6)	
H32	0.4371	0.4847	0.1530	0.039*	
C33	0.38214 (15)	0.2367 (4)	0.17282 (7)	0.0312 (6)	
C34	0.44284 (16)	0.1363 (4)	0.19169 (8)	0.0359 (6)	
H34	0.4886	0.1961	0.1908	0.043*	
C35	0.43683 (17)	−0.0466 (4)	0.21127 (8)	0.0391 (7)	
H35	0.4785	−0.1126	0.2237	0.047*	
C36	0.31342 (17)	−0.0411 (4)	0.19604 (9)	0.0414 (7)	
H36	0.2684	−0.1037	0.1978	0.050*	
C37	0.31675 (16)	0.1414 (4)	0.17617 (9)	0.0391 (6)	
H37	0.2740	0.2045	0.1645	0.047*	
C38	0.3673 (2)	−0.3277 (4)	0.23577 (9)	0.0507 (9)	
H38C	0.3644	−0.2941	0.2635	0.076*	
H38B	0.4093	−0.4140	0.2337	0.076*	
H38A	0.3243	−0.4035	0.2251	0.076*	
C39	0.10720 (14)	1.0136 (4)	0.06567 (8)	0.0315 (6)	
C40	0.12965 (14)	1.1045 (4)	0.10139 (8)	0.0324 (6)	
H40	0.1430	1.2466	0.1029	0.039*	
C41	0.13245 (14)	0.9851 (4)	0.13503 (8)	0.0320 (6)	
H41	0.1470	1.0456	0.1600	0.038*	
C42	0.11398 (13)	0.7763 (4)	0.13242 (8)	0.0282 (5)	
C43	0.09127 (14)	0.6893 (4)	0.09603 (8)	0.0334 (6)	
H43	0.0784	0.5469	0.0943	0.040*	
C44	0.08723 (15)	0.8083 (4)	0.06225 (8)	0.0353 (6)	
H44	0.0711	0.7500	0.0373	0.042*	

### Atomic displacement parameters (Å^2^)

**Table:**

	*U*^11^	*U*^22^	*U*^33^	*U*^12^	*U*^13^	*U*^23^
S1	0.0308 (3)	0.0233 (3)	0.0340 (4)	0.0026 (2)	0.0037 (3)	−0.0017 (2)
S2	0.0372 (4)	0.0287 (3)	0.0351 (4)	−0.0001 (3)	0.0014 (3)	0.0070 (3)
O8	0.120 (7)	0.037 (3)	0.031 (3)	−0.006 (4)	−0.005 (4)	0.005 (3)
O9	0.025 (4)	0.170 (10)	0.084 (7)	−0.015 (5)	0.010 (4)	0.071 (7)
O10	0.065 (6)	0.073 (5)	0.046 (4)	0.034 (4)	−0.003 (4)	0.017 (4)
O8'	0.135 (5)	0.039 (2)	0.036 (2)	0.014 (3)	0.030 (3)	0.0050 (16)
O9'	0.065 (3)	0.048 (2)	0.044 (3)	−0.024 (2)	0.007 (2)	0.0114 (18)
O10'	0.027 (2)	0.133 (5)	0.095 (5)	−0.007 (3)	−0.013 (2)	0.081 (4)
O1	0.0700 (17)	0.0481 (14)	0.0498 (13)	0.0011 (12)	0.0087 (12)	−0.0167 (11)
O2	0.117 (2)	0.0632 (16)	0.0299 (12)	−0.0117 (16)	0.0045 (13)	0.0008 (11)
O3	0.120 (2)	0.0370 (12)	0.0332 (11)	0.0231 (13)	0.0045 (13)	−0.0002 (9)
O4	0.0716 (17)	0.0579 (14)	0.0557 (14)	−0.0352 (13)	0.0181 (12)	−0.0154 (12)
O5	0.0406 (13)	0.0849 (18)	0.0732 (17)	0.0226 (13)	−0.0017 (12)	−0.0396 (15)
O6	0.158 (3)	0.0651 (17)	0.0270 (12)	0.0008 (18)	0.0034 (15)	0.0041 (12)
O7	0.0759 (17)	0.0416 (12)	0.0488 (13)	0.0006 (11)	0.0133 (12)	0.0152 (10)
N1	0.0300 (12)	0.0328 (12)	0.0405 (13)	−0.0008 (9)	0.0020 (10)	−0.0117 (10)
N2	0.0545 (15)	0.0242 (11)	0.0278 (11)	−0.0015 (10)	0.0024 (10)	−0.0011 (9)
N3	0.0448 (15)	0.0451 (14)	0.0363 (14)	−0.0143 (12)	0.0102 (11)	−0.0080 (11)
N4	0.0316 (12)	0.0329 (12)	0.0387 (13)	0.0022 (9)	0.0026 (10)	0.0096 (10)
N5	0.0617 (17)	0.0256 (11)	0.0256 (11)	0.0021 (11)	0.0028 (11)	0.0009 (9)
N6	0.0508 (16)	0.0431 (14)	0.0321 (13)	0.0114 (12)	0.0114 (11)	0.0069 (11)
C1	0.0401 (16)	0.0323 (14)	0.0466 (17)	0.0056 (12)	0.0058 (13)	−0.0091 (12)
C2	0.0424 (17)	0.0389 (15)	0.0484 (17)	−0.0017 (13)	−0.0019 (14)	−0.0175 (14)
C3	0.0274 (13)	0.0276 (12)	0.0285 (13)	−0.0014 (10)	0.0054 (10)	−0.0009 (10)
C4	0.0233 (13)	0.0376 (14)	0.0453 (16)	−0.0023 (11)	0.0003 (11)	−0.0083 (12)
C5	0.0301 (14)	0.0354 (14)	0.0462 (16)	−0.0002 (11)	0.0058 (12)	−0.0062 (12)
C6	0.0301 (13)	0.0247 (12)	0.0321 (13)	−0.0012 (10)	0.0072 (10)	−0.0012 (10)
C7	0.0258 (13)	0.0320 (13)	0.0301 (13)	−0.0021 (10)	0.0021 (10)	−0.0033 (11)
C8	0.0237 (13)	0.0341 (13)	0.0340 (14)	0.0002 (10)	0.0029 (10)	−0.0050 (11)
C9	0.0300 (13)	0.0282 (12)	0.0343 (14)	0.0024 (10)	0.0060 (11)	−0.0004 (11)
C10	0.0344 (14)	0.0280 (12)	0.0324 (14)	0.0003 (11)	0.0054 (11)	−0.0015 (11)
C11	0.0352 (14)	0.0280 (12)	0.0278 (13)	−0.0020 (11)	0.0033 (11)	0.0016 (10)
C12	0.0356 (15)	0.0274 (13)	0.0431 (16)	−0.0016 (11)	0.0046 (12)	−0.0052 (11)
C13	0.0420 (16)	0.0325 (14)	0.0367 (15)	0.0023 (12)	0.0036 (12)	−0.0016 (12)
C14	0.0473 (17)	0.0336 (14)	0.0345 (15)	−0.0111 (13)	−0.0002 (12)	−0.0023 (12)
C15	0.0392 (15)	0.0370 (14)	0.0299 (14)	−0.0049 (12)	0.0024 (11)	0.0004 (11)
C16	0.081 (2)	0.0283 (14)	0.0349 (16)	0.0015 (15)	0.0043 (15)	−0.0068 (12)
C17	0.0319 (14)	0.0359 (14)	0.0308 (14)	−0.0062 (11)	0.0071 (11)	−0.0045 (11)
C18	0.0368 (15)	0.0358 (14)	0.0337 (14)	−0.0048 (12)	0.0021 (12)	0.0055 (12)
C19	0.0336 (14)	0.0279 (13)	0.0389 (15)	0.0003 (11)	0.0005 (11)	0.0041 (11)
C20	0.0249 (12)	0.0281 (12)	0.0331 (13)	−0.0006 (10)	0.0062 (10)	−0.0010 (10)
C21	0.0328 (14)	0.0277 (12)	0.0329 (14)	−0.0011 (11)	0.0014 (11)	0.0024 (11)
C22	0.0337 (14)	0.0285 (13)	0.0378 (15)	−0.0012 (11)	0.0068 (11)	−0.0026 (11)
C23	0.0427 (18)	0.0420 (16)	0.0532 (19)	0.0034 (13)	−0.0023 (14)	0.0195 (14)
C24	0.0399 (16)	0.0344 (14)	0.0424 (16)	−0.0011 (12)	0.0054 (13)	0.0073 (12)
C25	0.0291 (13)	0.0276 (12)	0.0294 (13)	0.0047 (10)	0.0037 (10)	0.0014 (10)
C26	0.0283 (13)	0.0297 (13)	0.0364 (14)	0.0004 (10)	0.0016 (11)	0.0016 (11)
C27	0.0262 (13)	0.0308 (13)	0.0318 (13)	0.0064 (10)	0.0003 (10)	0.0012 (11)
C28	0.0320 (14)	0.0260 (12)	0.0309 (13)	0.0024 (10)	0.0046 (11)	0.0019 (10)
C29	0.0265 (13)	0.0361 (14)	0.0418 (15)	0.0003 (11)	0.0007 (11)	0.0070 (12)
C30	0.0252 (13)	0.0381 (14)	0.0385 (15)	0.0045 (11)	−0.0028 (11)	0.0088 (12)
C31	0.0297 (14)	0.0290 (13)	0.0356 (14)	0.0025 (10)	0.0041 (11)	0.0010 (11)
C32	0.0359 (15)	0.0295 (13)	0.0331 (14)	0.0023 (11)	0.0053 (11)	0.0023 (11)
C33	0.0408 (15)	0.0278 (12)	0.0248 (12)	0.0024 (11)	0.0033 (11)	0.0011 (10)
C34	0.0414 (16)	0.0346 (14)	0.0313 (14)	0.0054 (12)	0.0021 (12)	0.0046 (11)
C35	0.0517 (18)	0.0339 (14)	0.0309 (14)	0.0076 (13)	0.0023 (13)	0.0020 (12)
C36	0.0460 (17)	0.0357 (15)	0.0414 (16)	−0.0033 (13)	0.0013 (13)	0.0040 (13)
C37	0.0417 (16)	0.0344 (14)	0.0408 (16)	−0.0002 (12)	0.0029 (13)	0.0052 (12)
C38	0.089 (3)	0.0282 (14)	0.0342 (16)	−0.0011 (15)	0.0041 (16)	0.0071 (12)
C39	0.0306 (14)	0.0339 (13)	0.0308 (13)	0.0078 (11)	0.0074 (11)	0.0047 (11)
C40	0.0372 (15)	0.0249 (12)	0.0356 (14)	0.0021 (11)	0.0058 (11)	0.0010 (11)
C41	0.0365 (15)	0.0277 (13)	0.0309 (13)	0.0025 (11)	0.0004 (11)	0.0012 (10)
C42	0.0229 (12)	0.0266 (12)	0.0348 (13)	0.0037 (10)	0.0027 (10)	0.0024 (10)
C43	0.0314 (14)	0.0268 (12)	0.0406 (15)	−0.0009 (11)	−0.0012 (11)	−0.0024 (11)
C44	0.0374 (15)	0.0367 (14)	0.0306 (14)	0.0057 (12)	−0.0002 (11)	−0.0047 (11)

### Geometric parameters (Å, º)

**Table:**

S1—O4	1.430 (2)	C15—H15	0.9500
S1—O5	1.430 (2)	C16—H16B	0.9800
S1—O3	1.431 (2)	C16—H16C	0.9800
S1—C20	1.786 (3)	C16—H16A	0.9800
S2—O9	1.389 (5)	C17—C22	1.379 (4)
S2—O10'	1.395 (4)	C17—C18	1.383 (4)
S2—O9'	1.440 (3)	C18—C19	1.383 (4)
S2—O8'	1.442 (3)	C18—H18	0.9500
S2—O8	1.443 (5)	C19—C20	1.388 (4)
S2—O10	1.443 (5)	C19—H19	0.9500
S2—C42	1.789 (3)	C20—C21	1.387 (3)
O1—N3	1.225 (3)	C21—C22	1.384 (4)
O2—N3	1.215 (3)	C21—H21	0.9500
O6—N6	1.207 (3)	C22—H22	0.9500
O7—N6	1.220 (3)	C23—H23A	0.9800
N1—C3	1.364 (3)	C23—H23C	0.9800
N1—C1	1.440 (3)	C23—H23B	0.9800
N1—C2	1.446 (3)	C24—H24B	0.9800
N2—C13	1.341 (4)	C24—H24C	0.9800
N2—C14	1.345 (4)	C24—H24A	0.9800
N2—C16	1.476 (3)	C25—C30	1.402 (4)
N3—C17	1.473 (3)	C25—C26	1.408 (3)
N4—C25	1.369 (3)	C26—C27	1.374 (3)
N4—C23	1.440 (3)	C26—H26	0.9500
N4—C24	1.448 (3)	C27—C28	1.398 (4)
N5—C35	1.343 (4)	C27—H27	0.9500
N5—C36	1.345 (4)	C28—C29	1.395 (4)
N5—C38	1.480 (3)	C28—C31	1.453 (3)
N6—C39	1.473 (3)	C29—C30	1.382 (4)
C1—H1A	0.9800	C29—H29	0.9500
C1—H1C	0.9800	C30—H30	0.9500
C1—H1B	0.9800	C31—C32	1.341 (4)
C2—H2B	0.9800	C31—H31	0.9500
C2—H2C	0.9800	C32—C33	1.449 (3)
C2—H2A	0.9800	C32—H32	0.9500
C3—C4	1.404 (3)	C33—C37	1.397 (4)
C3—C8	1.410 (3)	C33—C34	1.406 (4)
C4—C5	1.376 (4)	C34—C35	1.368 (4)
C4—H4	0.9500	C34—H34	0.9500
C5—C6	1.397 (4)	C35—H35	0.9500
C5—H5	0.9500	C36—C37	1.365 (4)
C6—C7	1.398 (3)	C36—H36	0.9500
C6—C9	1.454 (3)	C37—H37	0.9500
C7—C8	1.375 (3)	C38—H38C	0.9800
C7—H7	0.9500	C38—H38B	0.9800
C8—H8	0.9500	C38—H38A	0.9800
C9—C10	1.339 (4)	C39—C40	1.377 (4)
C9—H9	0.9500	C39—C44	1.378 (4)
C10—C11	1.452 (3)	C40—C41	1.381 (4)
C10—H10	0.9500	C40—H40	0.9500
C11—C12	1.394 (4)	C41—C42	1.392 (3)
C11—C15	1.405 (4)	C41—H41	0.9500
C12—C13	1.366 (4)	C42—C43	1.387 (4)
C12—H12	0.9500	C43—C44	1.382 (4)
C13—H13	0.9500	C43—H43	0.9500
C14—C15	1.372 (4)	C44—H44	0.9500
C14—H14	0.9500		
			
O4—S1—O5	113.13 (17)	H16B—C16—H16C	109.5
O4—S1—O3	113.31 (17)	N2—C16—H16A	109.5
O5—S1—O3	113.42 (17)	H16B—C16—H16A	109.5
O4—S1—C20	104.38 (13)	H16C—C16—H16A	109.5
O5—S1—C20	105.70 (13)	C22—C17—C18	122.8 (2)
O3—S1—C20	105.87 (12)	C22—C17—N3	118.3 (3)
O9—S2—O10'	135.7 (5)	C18—C17—N3	119.0 (3)
O9—S2—O9'	34.6 (5)	C17—C18—C19	118.2 (3)
O10'—S2—O9'	113.6 (4)	C17—C18—H18	120.9
O9—S2—O8'	75.0 (6)	C19—C18—H18	120.9
O10'—S2—O8'	117.6 (4)	C18—C19—C20	120.3 (2)
O9'—S2—O8'	109.5 (3)	C18—C19—H19	119.9
O9—S2—O8	119.2 (6)	C20—C19—H19	119.9
O10'—S2—O8	71.0 (4)	C21—C20—C19	120.3 (2)
O9'—S2—O8	147.7 (3)	C21—C20—S1	120.0 (2)
O8'—S2—O8	49.1 (4)	C19—C20—S1	119.6 (2)
O9—S2—O10	112.2 (7)	C22—C21—C20	120.1 (3)
O10'—S2—O10	35.9 (3)	C22—C21—H21	119.9
O9'—S2—O10	81.2 (5)	C20—C21—H21	119.9
O8'—S2—O10	148.7 (4)	C17—C22—C21	118.4 (3)
O8—S2—O10	106.6 (5)	C17—C22—H22	120.8
O9—S2—C42	110.8 (4)	C21—C22—H22	120.8
O10'—S2—C42	106.8 (2)	N4—C23—H23A	109.5
O9'—S2—C42	104.1 (2)	N4—C23—H23C	109.5
O8'—S2—C42	103.90 (18)	H23A—C23—H23C	109.5
O8—S2—C42	104.8 (3)	N4—C23—H23B	109.5
O10—S2—C42	101.6 (3)	H23A—C23—H23B	109.5
C3—N1—C1	121.2 (2)	H23C—C23—H23B	109.5
C3—N1—C2	120.5 (2)	N4—C24—H24B	109.5
C1—N1—C2	118.0 (2)	N4—C24—H24C	109.5
C13—N2—C14	119.8 (2)	H24B—C24—H24C	109.5
C13—N2—C16	119.4 (3)	N4—C24—H24A	109.5
C14—N2—C16	120.8 (3)	H24B—C24—H24A	109.5
O2—N3—O1	123.7 (3)	H24C—C24—H24A	109.5
O2—N3—C17	118.1 (3)	N4—C25—C30	121.7 (2)
O1—N3—C17	118.2 (3)	N4—C25—C26	121.7 (2)
C25—N4—C23	120.3 (2)	C30—C25—C26	116.6 (2)
C25—N4—C24	120.7 (2)	C27—C26—C25	121.6 (2)
C23—N4—C24	117.9 (2)	C27—C26—H26	119.2
C35—N5—C36	120.2 (2)	C25—C26—H26	119.2
C35—N5—C38	120.0 (3)	C26—C27—C28	121.8 (2)
C36—N5—C38	119.7 (3)	C26—C27—H27	119.1
O6—N6—O7	123.3 (3)	C28—C27—H27	119.1
O6—N6—C39	118.2 (3)	C29—C28—C27	116.6 (2)
O7—N6—C39	118.5 (3)	C29—C28—C31	120.5 (2)
N1—C1—H1A	109.5	C27—C28—C31	122.8 (2)
N1—C1—H1C	109.5	C30—C29—C28	122.1 (3)
H1A—C1—H1C	109.5	C30—C29—H29	119.0
N1—C1—H1B	109.5	C28—C29—H29	119.0
H1A—C1—H1B	109.5	C29—C30—C25	121.2 (2)
H1C—C1—H1B	109.5	C29—C30—H30	119.4
N1—C2—H2B	109.5	C25—C30—H30	119.4
N1—C2—H2C	109.5	C32—C31—C28	126.0 (3)
H2B—C2—H2C	109.5	C32—C31—H31	117.0
N1—C2—H2A	109.5	C28—C31—H31	117.0
H2B—C2—H2A	109.5	C31—C32—C33	125.5 (3)
H2C—C2—H2A	109.5	C31—C32—H32	117.3
N1—C3—C4	121.7 (2)	C33—C32—H32	117.3
N1—C3—C8	121.6 (2)	C37—C33—C34	116.2 (2)
C4—C3—C8	116.7 (2)	C37—C33—C32	124.5 (3)
C5—C4—C3	121.3 (3)	C34—C33—C32	119.3 (3)
C5—C4—H4	119.4	C35—C34—C33	120.8 (3)
C3—C4—H4	119.4	C35—C34—H34	119.6
C4—C5—C6	122.1 (3)	C33—C34—H34	119.6
C4—C5—H5	118.9	N5—C35—C34	120.9 (3)
C6—C5—H5	118.9	N5—C35—H35	119.6
C5—C6—C7	116.5 (2)	C34—C35—H35	119.6
C5—C6—C9	120.1 (2)	N5—C36—C37	121.1 (3)
C7—C6—C9	123.4 (2)	N5—C36—H36	119.5
C8—C7—C6	122.0 (2)	C37—C36—H36	119.5
C8—C7—H7	119.0	C36—C37—C33	120.9 (3)
C6—C7—H7	119.0	C36—C37—H37	119.5
C7—C8—C3	121.3 (2)	C33—C37—H37	119.5
C7—C8—H8	119.4	N5—C38—H38C	109.5
C3—C8—H8	119.4	N5—C38—H38B	109.5
C10—C9—C6	126.5 (2)	H38C—C38—H38B	109.5
C10—C9—H9	116.8	N5—C38—H38A	109.5
C6—C9—H9	116.8	H38C—C38—H38A	109.5
C9—C10—C11	124.7 (2)	H38B—C38—H38A	109.5
C9—C10—H10	117.7	C40—C39—C44	122.5 (2)
C11—C10—H10	117.7	C40—C39—N6	118.4 (2)
C12—C11—C15	116.7 (2)	C44—C39—N6	119.1 (2)
C12—C11—C10	123.9 (2)	C39—C40—C41	118.7 (2)
C15—C11—C10	119.4 (2)	C39—C40—H40	120.6
C13—C12—C11	120.5 (3)	C41—C40—H40	120.6
C13—C12—H12	119.7	C40—C41—C42	120.1 (3)
C11—C12—H12	119.7	C40—C41—H41	120.0
N2—C13—C12	121.6 (3)	C42—C41—H41	120.0
N2—C13—H13	119.2	C43—C42—C41	119.9 (2)
C12—C13—H13	119.2	C43—C42—S2	120.4 (2)
N2—C14—C15	121.1 (3)	C41—C42—S2	119.7 (2)
N2—C14—H14	119.4	C44—C43—C42	120.5 (2)
C15—C14—H14	119.4	C44—C43—H43	119.8
C14—C15—C11	120.3 (3)	C42—C43—H43	119.8
C14—C15—H15	119.9	C39—C44—C43	118.4 (3)
C11—C15—H15	119.9	C39—C44—H44	120.8
N2—C16—H16B	109.5	C43—C44—H44	120.8
N2—C16—H16C	109.5		
			
C1—N1—C3—C4	−179.6 (3)	C24—N4—C25—C26	7.8 (4)
C2—N1—C3—C4	−6.0 (4)	N4—C25—C26—C27	177.8 (2)
C1—N1—C3—C8	1.1 (4)	C30—C25—C26—C27	−0.6 (4)
C2—N1—C3—C8	174.7 (3)	C25—C26—C27—C28	−0.6 (4)
N1—C3—C4—C5	−176.7 (3)	C26—C27—C28—C29	1.3 (4)
C8—C3—C4—C5	2.7 (4)	C26—C27—C28—C31	−179.7 (2)
C3—C4—C5—C6	−1.5 (4)	C27—C28—C29—C30	−0.9 (4)
C4—C5—C6—C7	−0.5 (4)	C31—C28—C29—C30	−179.9 (2)
C4—C5—C6—C9	179.5 (3)	C28—C29—C30—C25	−0.3 (4)
C5—C6—C7—C8	1.3 (4)	N4—C25—C30—C29	−177.4 (2)
C9—C6—C7—C8	−178.7 (2)	C26—C25—C30—C29	1.1 (4)
C6—C7—C8—C3	0.0 (4)	C29—C28—C31—C32	175.3 (3)
N1—C3—C8—C7	177.4 (2)	C27—C28—C31—C32	−3.6 (4)
C4—C3—C8—C7	−1.9 (4)	C28—C31—C32—C33	179.5 (2)
C5—C6—C9—C10	174.7 (3)	C31—C32—C33—C37	−6.0 (4)
C7—C6—C9—C10	−5.3 (4)	C31—C32—C33—C34	173.2 (3)
C6—C9—C10—C11	−179.7 (2)	C37—C33—C34—C35	1.4 (4)
C9—C10—C11—C12	−9.8 (4)	C32—C33—C34—C35	−177.9 (2)
C9—C10—C11—C15	170.2 (3)	C36—N5—C35—C34	−0.6 (4)
C15—C11—C12—C13	−1.2 (4)	C38—N5—C35—C34	−177.8 (2)
C10—C11—C12—C13	178.9 (2)	C33—C34—C35—N5	−0.4 (4)
C14—N2—C13—C12	0.4 (4)	C35—N5—C36—C37	0.6 (4)
C16—N2—C13—C12	178.2 (2)	C38—N5—C36—C37	177.8 (3)
C11—C12—C13—N2	0.4 (4)	N5—C36—C37—C33	0.4 (4)
C13—N2—C14—C15	−0.4 (4)	C34—C33—C37—C36	−1.4 (4)
C16—N2—C14—C15	−178.1 (2)	C32—C33—C37—C36	177.9 (3)
N2—C14—C15—C11	−0.5 (4)	O6—N6—C39—C40	−179.0 (3)
C12—C11—C15—C14	1.2 (4)	O7—N6—C39—C40	0.0 (4)
C10—C11—C15—C14	−178.8 (2)	O6—N6—C39—C44	−0.4 (4)
O2—N3—C17—C22	−179.7 (3)	O7—N6—C39—C44	178.7 (3)
O1—N3—C17—C22	−1.5 (4)	C44—C39—C40—C41	0.2 (4)
O2—N3—C17—C18	0.0 (4)	N6—C39—C40—C41	178.8 (2)
O1—N3—C17—C18	178.1 (3)	C39—C40—C41—C42	1.1 (4)
C22—C17—C18—C19	0.3 (4)	C40—C41—C42—C43	−1.4 (4)
N3—C17—C18—C19	−179.4 (2)	C40—C41—C42—S2	177.7 (2)
C17—C18—C19—C20	−0.7 (4)	O9—S2—C42—C43	−56.2 (7)
C18—C19—C20—C21	1.2 (4)	O10'—S2—C42—C43	99.9 (4)
C18—C19—C20—S1	−177.6 (2)	O9'—S2—C42—C43	−20.6 (3)
O4—S1—C20—C21	−116.7 (2)	O8'—S2—C42—C43	−135.1 (4)
O5—S1—C20—C21	123.7 (2)	O8—S2—C42—C43	174.1 (5)
O3—S1—C20—C21	3.1 (3)	O10—S2—C42—C43	63.2 (5)
O4—S1—C20—C19	62.1 (3)	O9—S2—C42—C41	124.7 (7)
O5—S1—C20—C19	−57.5 (3)	O10'—S2—C42—C41	−79.2 (4)
O3—S1—C20—C19	−178.1 (2)	O9'—S2—C42—C41	160.3 (3)
C19—C20—C21—C22	−1.3 (4)	O8'—S2—C42—C41	45.8 (4)
S1—C20—C21—C22	177.5 (2)	O8—S2—C42—C41	−5.0 (5)
C18—C17—C22—C21	−0.3 (4)	O10—S2—C42—C41	−115.9 (5)
N3—C17—C22—C21	179.4 (2)	C41—C42—C43—C44	0.4 (4)
C20—C21—C22—C17	0.8 (4)	S2—C42—C43—C44	−178.7 (2)
C23—N4—C25—C30	−5.9 (4)	C40—C39—C44—C43	−1.2 (4)
C24—N4—C25—C30	−173.8 (3)	N6—C39—C44—C43	−179.8 (2)
C23—N4—C25—C26	175.8 (3)	C42—C43—C44—C39	0.9 (4)
